# A Hypertrophic Spinal Pachymeningitis Patient With Factor V Leiden (G1691A), MTHFR C677T, MTHFR A1298C, PAI-1 4G-5G, Glycoprotein IIIa L33P Gene Mutations

**DOI:** 10.7759/cureus.29937

**Published:** 2022-10-05

**Authors:** Serkan Civlan, Cemre Harvey, Duygu Herek, İbrahim Türkçüer, Ramazan Sabirli, Matteo Pellegrini, Aylin Koseler

**Affiliations:** 1 Neurosurgery, Pamukkale University Faculty of Medicine, Denizli, TUR; 2 Emergency Medicine, Arnavutkoy State Hospital, Istanbul, TUR; 3 Radiology, Pamukkale University Medical Faculty, Denizli, TUR; 4 Emergency, Pamukkale University Medical Faculty, Denizli, TUR; 5 Emergency, Izmir Bakircay University, Cigli Training and Research Hospital, Izmir, TUR; 6 Molecular, Cell and Developmental Biology, University of California Los Angeles David Geffen School of Medicine, Los Angeles, USA; 7 Biophysics, Pamukkale University, Denizli, TUR

**Keywords:** glycoprotein iiia l33p gene, pai-1 4g-5g, mthfr a1298c, mthfr c677t, factor v leiden (g1691a), hypertrophic spinal pachymeningitidis

## Abstract

Hypertrophic pachymeningitis (HP) is a rare clinical entity of diverse etiology, characterized by a chronic inflammation that causes dura thickening. Reports of Idiopathic hypertrophic cranial pachymeningitis (IHCP) were related to infections, trauma, tumors, and rheumatologic conditions. It was first described by Charcot and Joffroy regarding spinal meninges in 1869. HP has three stages; progressive radicular symptoms begin first, then muscle weakness and atrophy start. Findings such as paraplegia, loss of bladder and bowel control, and respiratory distress caused by intercostal and diaphragmatic denervation are considered the third stage of the disease. Especially in the cranial form of the disease, nerve ischemia and various cranial neuropathic findings may occur.

Factor V Leiden (G1691A), MTHFR C677T, MTHFR A1298C, and PAI-1 4G-5G gene mutation analysis were measured with an ABI Prism. In this case report, the authors present a case of hypertrophic mutations pachymeningitis with Factor V Leiden (G1691A), MTHFR C677T, MTHFR A1298C, PAI-1 4G-5G, Glycoprotein IIIa L33P gene.

In conclusion, we report a case of HP with Factor V Leiden (G1691A), MTHFR C677T, MTHFR A1298C, PAI-1 4G-5G, and Glycoprotein IIIa L33P gene mutations. We emphasize that the identification of pachymeningitis can be easily bypassed with the application of limited laboratory techniques. As in this case report, we think that these mutations should be analyzed in patients diagnosed with pachymeningitis.

## Introduction

Hypertrophic pachymeningitis (HP) is a rare clinical entity of diverse etiology, characterized by a chronic inflammation that causes dura thickening. Reports of Idiopathic hypertrophic cranial pachymeningitis (IHCP) were related to infections, trauma, tumors, and rheumatologic conditions [1]. Charcot and Joffroy described firstly regarding spinal meninges in 1869 [2]. HP has three stages - progressive radicular symptoms begin first, then muscle weakness and atrophy start. Findings such as paraplegia, loss of bladder and bowel control, and respiratory distress caused by intercostal and diaphragmatic denervation are considered the third stage of the disease. Especially in the cranial form of the disease, nerve ischemia and various cranial neuropathic findings may occur [3]. The authors present a case of hypertrophic mutations pachymeningitis with Factor V Leiden (G1691A), MTHFR C677T, MTHFR A1298C, PAI-1 4G-5G, Glycoprotein IIIa L33P gene.

## Case presentation

A 62-year-old woman visited the clinic with back pain and bilateral numbness in her legs after a slipping fall. There are two traffic accidents and a couple of fractures in her femoral bones in her history. She stated that she has had pain and numbness in her lower extremities for years after the accident. In addition, she has had one abortion in her history.

A bilateral lower extremity physical examination consisted of normal vital findings and 5/5 muscle strength, intact and equal pulses, and equal bilateral diameters. She had neuropathic complaints in her bilateral lower extremities. Other parameters and findings were in the normal range (Tables 1, 2).

**Table 1:** Biochemical parameters of the patient GFR, glomerular filtration ratio; CRP, C-reactive protein; AST, aspartate aminotransferase; ALT, alanine aminotransferase; LDH, lactate dehydrogenase

Laboratory Parameter	Level	Unit	Normal Range
GFR	91	mL/min	>90
Glucose	97	mg/dL	82-115
Urea	51	mg/dL	<50
Creatinine	0.72	mg/dL	0.5-0.95
Na^+1^	143	mmol/L	136-145
Cl^-^	105	mmol/L	98-1,047
K^-^	5.27	mmol/L	3.5-5.1
Total Protein	60.5	g/L	66-87
Albumin	37.5	g/L	35-52
AST	28	IU/L	<32
ALT	19	IU/L	<33
LDH	214	IU/L	135-214
Ca^+^^2^	8.65	mg/dL	8.8-10.2
CRP	4.33	mg/dL	0.5-5
Homocystein	16.45	µmol/L	5-12

**Table 2:** Hematological parameters and coagulation parameters of the patient Neu, neutrophil; LYM, lymphocyte; Mono, monocyte; Eo, eosinophil; RBC, red blood cell; MCV, mean corpuscular volume; MCH, mean corpuscular hemoglobin; MCHC, mean corpuscular hemoglobin concentration; RDW, red cell distribution width; NLR, neutrophil to lymphocyte ratio; APTT, activated partial thromboplastin time; PTT, prothrombin time; INR, international normalized ratio.

Laboratory Parameter	Level	Unit	Normal Range
WBC Count	5.41	K/µL	4-10
%NEU	56,2	%	50-70
NEU Count	3.05	K/µL	2-7
% LYM	33,9	%	20-40
Monocyte Count	0.27	K/µL	0.12-1.20
%Mono	5	%	3-12
Eosinophil Count	0.24	K/µL	0.02-0.5
%Eo	4.5	%	0.5-5
Haemoglobin	11.7	g/dL	11-16
RBC	3.75	M/µL	3.5-5.5
Hematocrit	35.1	%	37-54
MCV	93.8	fL	80-100
MCH	31.1	pg	27-34
MCHC	33.2	g/dL	32-36
Platelet	190	K/µL	150-300
RDW	13.5	%	11.0-16.0
NLR	1.67		0-3.13
APTT	29,7	Second	26-35
PTT	10,5	Second	10.2-14.4
INR	0.9		0.85-1.2

For a DVT prediagnosis, rheumatism parameters and a thrombophilia genetic panel were collected in her previous visits. She has been administered Gabapentin, acetylsalicylic acid, metformin, and Vitamin B. Among the hematological and biochemical parameters of the patient, only the homocysteine level was high. (Tables 1, 2). Protein C, Protein S, and activated Protein C resistance levels and all rheumatologic parameters were found within the normal range (Table 3).

**Table 3:** Rheumatological and thrombophilia parameters of the patient

Laboratory Parameter	Level	Unit	Normal Range
Indirect Coombs	Negative		
Indirect Coombs (with enzyme)	Negative		
Direct Coombs IgG	Negative		
Direct Coombs Complement (C3d)	Negative		
Anti Histone Antibody	Negative		
Nucleosome	Negative		
Anti Ribosomal P Protein	Negative		
Anti-Sm/Rnp (Immonoblotting)	Negative		
Anti-Ssa	Negative		
Anti -Jo 1 (Immunoblotting)	Negative		
Anti -Ssb	Negative		
Anti -Scl 70	Negative		
Anti -Sm (Immonoblotting)	Negative		
Anti Nuclear Antibody-Ana	Negative		
Anti Beta-2 Glycoprotein 1 IgG	1.53 Negative	U/Ml	0-5
Anti Beta-2 Glycoprotein 1 IgM	0.79 Negative	U/Ml	0-5
Anti Beta-2 Glycoprotein 1 IgA	1.39 Negative	U/Ml	0-5
Anti Phospholipid IgM	1.46 Negative	Gpl Au/Ml	0-10
Anti Phospholipid IgG	2.82 Negative	Gpl Au/Ml	0-10
Antı Cardiolipin IgG	0.22 Negative	Gpl Au/Ml	0-10
Antı Cardiolipi IgM	3.41 Negative	Mpl U/Ml	0-10
Protein C	130	%	70-140
Protein S	110.6	%	54.7-123.7
Activated protein resistance	1.85	%	

**Figure 1:**
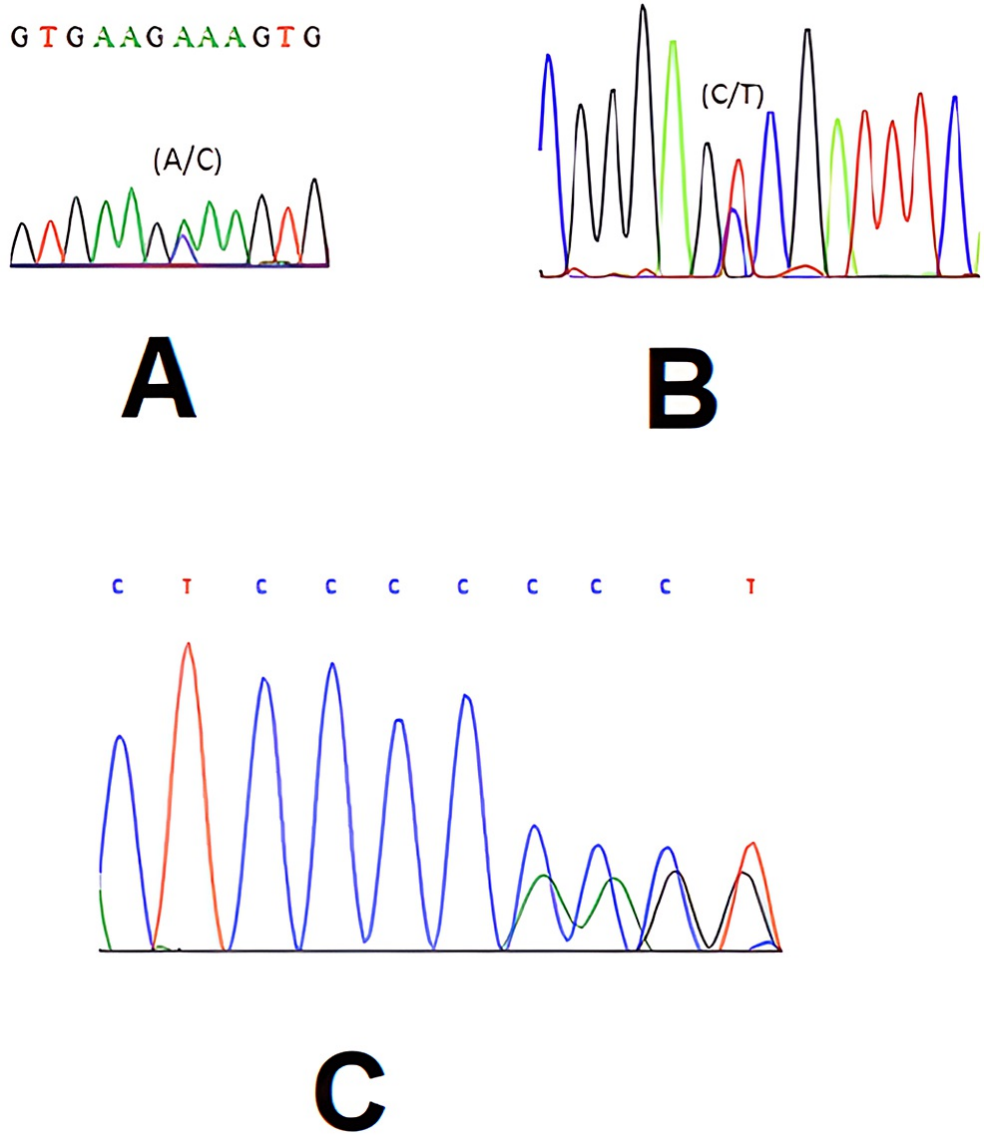
DNA sequencing (A) Sequence for MTHFR A1298C mutation, (B) sequence for MTHFR C677T mutation, (C) sequence for PAI-1 4G-5G mutation

Factor V Leiden (G1691A) and Glycoprotein IIIa L33P mutations were done with real-time PCR analysis. MTHFR C677T, MTHFR A1298C, and PAI-1 4G-5G gene mutation analysis were determined with the ABI prism DNA sequencing system (Figure 1).

The individual was found to be Factor V Leiden G1691A heterozygous, MTHFR C677T heterozygous, MTHFR A1298C heterozygous, PAI-1 4G-5G heterozygous, glycoprotein IIIa L33P heterozygous.

**Figure 2:**
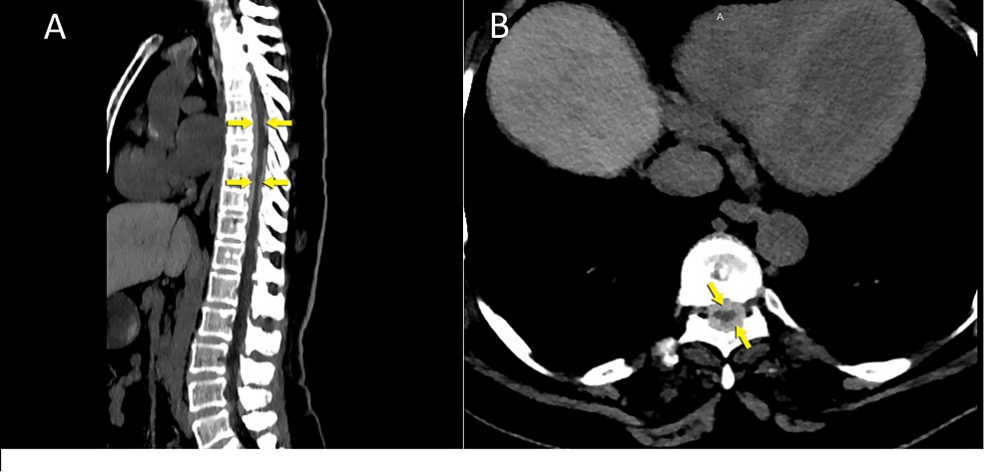
Thoracal segment of pachymeningitidis

CT scans showed diffuse hyperdense thickening in the dura from the T1 level to the T12 level in the spinal cord. An MRI was obtained due to significant thoracal spinal cord suppression due to the thickening (Figures 2A, 2B).

**Figure 3:**
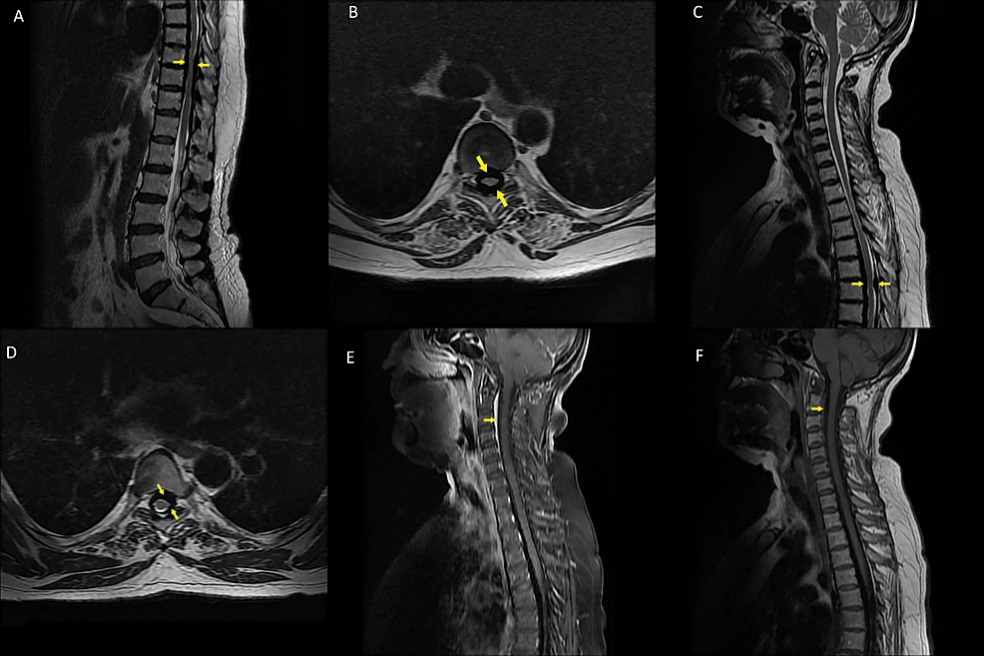
Thoracal and lumbar segments of pahcymeningitidis

Diffuse T1-2 hypointense thickening was observed in the dura starting from the T1 level and extending anteriorly to the T11 level and extending posteriorly from the T1 level to the L4-5 level (Figures 3A-3D). Thoracal dural thickening caused significant spinal cord compression. In some locations, myelomalasic signals in the spinal cord were observed. There was no contrast agent uptake in the thickening after intravenous contrast agent. However, intense contrast agent enhancement in the extradural area at the C2-3 level in the anterior and upper cervical left half of the spinal canal was seen (Figures 3E, 3F).

The patient was prediagnosed with IHCP. The patient was hospitalized and started Prednisolone treatment. Follow-up visits showed no neurologic deficits, and therefore the patient was discharged. MRI scans from a three-month follow-up showed no significant change.

## Discussion

In this study, Factor V Leiden (G1691A), MTHFR C677T, MTHFR A1298C, PAI-1 4G-5G, and Glycoprotein IIIa L33P gene mutations were identified by DNA sequence analysis. To the best of our knowledge, our case is the first reported case of pachymeningitis which has multiple mutations in hematological genes. The incidence of hypertrophic spinal pachymeningitidis was found to be higher in men than in women in a study (1:0.91) and this case is also important in terms of being an example of pachymeningitis in women [4]. Several proteases are involved in Factor V (FV) activation, including factor V, thrombin, factor Xa (FXa), and (transiently) plasmin. FXa and FVa form a prothrombinase complex, which increases the conversion rate of prothrombin to α-thrombin 300,000 times. Protein C (PC) inactivates FV, thereby regulating the amount of thrombin produced. It was found that the addition of activated protein C (APC) to the plasma of some patients with hereditary thrombophilia did not prolong the clotting time. To date, few FV gene mutations have been identified in association with APC resistance [5].

 As a result of the point mutation (Factor V Leiden Mutation [1691G>A]) in the factor V gene, glutamine replaces the arginine at 506 amino acid position. Mutated FV is inactivated 10 times slower than normal FV and remains in circulation for a longer period. This causes more thrombin production and mild hypercoagulation, reflecting the increase of factor XII and activated coagulation factors from prothrombin fragments. In this situation, the factor V molecule confers resistance to proteolytic inactivation of APC, and as a result, individuals carrying this mutation are more likely to develop venous thrombosis [6].

Homocysteine is a sulfurous amino acid that is formed by the removal of a methyl group from methionine and plays a regulatory role in remethylation and transsulfuration. metabolic pathways. In the remethylation pathway, homocysteine is methylated and converted back to methionine in a reaction in which vitamin B12 (cobalamin) is used as a cofactor and 5-methyltetrahydrofolate (MTHF) is used as a substrate, and the enzyme methionine synthase functions. It is synthesized from methylenetetrahydrofolate (derived from dietary folate) by a reaction catalyzed by the enzyme 5-MTHF thermolabile methylenetetrahydrofolate reductase (MTHFR), which is the substrate of this metabolic pathway, and therefore it is used in folic acid deficiencies. The amount of substrate required for the remethylation pathway also decreases [7].

It has been shown that the C677T homozygous polymorphism (TT) of the MTHFR enzyme slows down the enzyme activity, thus the activity of the remethylation cycle, and is associated with significantly higher homocysteine levels [8]. It has been shown that the A1298C polymorphism of the MTHFR enzyme also affects total homocysteine concentrations and is a risk factor for neural tube defects [9].

Glycoprotein IIb/IIIa receptors are proteins in the integrin class and are not found on the surface of circulating platelets but are presented on the surface by platelet activation. It is a calcium-dependent heterodimer and acts as a receptor for fibrinogen, fibronectin, vitronectin, vWF, and thrombospondin and governs platelet aggregation, tight adhesion, and scattering. There are studies on the glycoprotein IIb/IIIa polymorphism. Platelet antigen polymorphism in the form of PL(A1) and PL(A2) of the gene encoding GpIIIa is common and associated with vascular diseases [10]. Three polymorphisms of GPIIb/IIIa have been identified. A1/A2 (Leu33Pro), a single nucleotide polymorphism (SNP) at position 196 in the beta 3 integrin gene, is most thoroughly investigated for its potential pathophysiological role in CAD.

Plasminogen activator inhibitor-1 (PAI-1) is the best known of the activator molecules. PAI-1 is a protein from the serpin family and exerts its effect through t-PA inhibition. PAI-1, together with t-PA, binds to fibrin and exerts its inhibitory effect. Since the source of PAI 1 and t-PA is endothelial and vascular smooth muscle cells, fibrinolysis is locally controlled [11]. A common polymorphism, known as 4G/5G, a deletion/insertion polymorphism of single guanine in the promoter region of the PAI-1 gene at base-pair - 675, is found in homozygosity in approximately 25% of the general population [12].

## Conclusions

In conclusion, we report a case of HP with Factor V Leiden (G1691A), MTHFR C677T, MTHFR A1298C, PAI-1 4G-5G, Glycoprotein IIIa L33P gene mutations. We emphasize that genetic analysis may also be useful in the diagnosis of pachymeningitis and the determination of its cause, in addition to existing radiological examinations. As in this case report, we think that these mutations should be analyzed in patients diagnosed with pachymeningitis.
